# MatPred: Computational Identification of Mature MicroRNAs within Novel Pre-MicroRNAs

**DOI:** 10.1155/2015/546763

**Published:** 2015-11-23

**Authors:** Jin Li, Ying Wang, Lei Wang, Weixing Feng, Kuan Luan, Xuefeng Dai, Chengzhen Xu, Xianglian Meng, Qiushi Zhang, Hong Liang

**Affiliations:** ^1^Institute of Biomedical Engineering, College of Automation, Harbin Engineering University, 145 Nantong Street, Nangang District, Harbin, Heilongjiang 150001, China; ^2^Bioinformatics Research Center, College of Automation, Harbin Engineering University, 145 Nantong Street, Nangang District, Harbin, Heilongjiang 150001, China; ^3^Network Information Center, Qiqihar University, No. 42, Wenhua Street, Qiqihar, Heilongjiang 161006, China

## Abstract

*Background.* MicroRNAs (miRNAs) are short noncoding RNAs integral for regulating gene expression at the posttranscriptional level. However, experimental methods often fall short in finding miRNAs expressed at low levels or in specific tissues. While several computational methods have been developed for predicting the localization of mature miRNAs within the precursor transcript, the prediction accuracy requires significant improvement.* Methodology/Principal Findings.* Here, we present MatPred, which predicts mature miRNA candidates within novel pre-miRNA transcripts. In addition to the relative locus of the mature miRNA within the pre-miRNA hairpin loop and minimum free energy, we innovatively integrated features that describe the nucleotide-specific RNA secondary structure characteristics. In total, 94 features were extracted from the mature miRNA loci and flanking regions. The model was trained based on a radial basis function kernel/support vector machine (RBF/SVM). Our method can predict precise locations of mature miRNAs, as affirmed by experimentally verified human pre-miRNAs or pre-miRNAs candidates, thus achieving a significant advantage over existing methods.* Conclusions.* MatPred is a highly effective method for identifying mature miRNAs within novel pre-miRNA transcripts. Our model significantly outperformed three other widely used existing methods. Such processing prediction methods may provide important insight into miRNA biogenesis.

## 1. Introduction

MicroRNAs (miRNAs) are short (generally 19–27 nucleotides), single-stranded noncoding RNAs that regulate gene expression at the posttranscriptional level [1]. miRNAs play important roles in a variety of biological processes, including organism development, tissue differentiation, and cell cycle [2, 3]. Dysregulation of miRNA processing is associated with many diseases, including cancer [4].

The processes of miRNA biogenesis in animals have been extensively studied. During this process, the long primary miRNA transcripts (pri-miRNAs) are cropped into double-stranded precursor miRNAs (pre-miRNAs) by a microprocessor complex, including Drosha and its cofactor DGCR8/Pasha [5]. Pre-miRNAs are usually ~70 nt and have a typical hairpin structure with an overhang of 2 nt on their 3′ ends [6]. The pre-miRNAs are then exported from the nucleus by carrier proteins such as exportin-5 [7] and most are cleaved into ~22 bp miRNA:miRNA^*∗*^ duplexes through the endonuclease Dicer [8, 9]. The miRNA:miRNA^*∗*^ duplex usually has two stable strands. While previous studies suggested one strand is the mature miRNA (the “guide strand”) that is incorporated into the RNA-induced silencing complex (RISC) for mRNA transcript target recognition [10–12], more recent studies showed that both mature miRNA and mature miRNA^*∗*^ species may exhibit inhibitory activity [13]. Consequently, the biogenesis of miRNAs is a very complex process, and knowledge of its various components/mechanisms would be very beneficial to the field of molecular physiology [14, 15].

Current experimental techniques, such as cloning and constructing short RNA libraries [16–18], can be used to identify miRNAs, but they often cannot find miRNAs that are expressed at low levels or in specific tissues. Moreover, not all miRNAs can be cloned. Recently, the emergence of next-generation sequencing (NGS) technologies, such as deep-sequencing, makes it possible to localize tissue-specific and development stage-specific miRNAs on a genome-wide scale. As NGS methods generate hundreds of millions of reads, separating miRNAs from other small RNA species or RNA degradation products is challenging [19, 20]. Consequently, computational approaches need to be developed to complement experimental techniques.

Several computational approaches have been reported for identifying mature miRNAs from their pre-miRNA transcripts. For instance, MiRmat [21] was designed to predict Drosha and Dicer cleavage sites in vertebrates using a Random Forest method. MiRRim2 [22] is designed for predicting human mature miRNA candidates using a conditional random fields (CRFs) method, based on evolutionary conserved features upstream of Drosha cleavage sites. MatureBayes [23] uses a Naive Bayes classifier to identify the location of human and mouse mature miRNAs based on information specific to individual nucleotide. Microprocessor uses a support vector machine (SVM) [24] classifier to identify Drosha cleavage sites based on 686 sequence- and structure-related features. MaturePred [25] finds mature miRNAs by integrating the structure-based free energy, and other features extracted from miRNA:miRNA^*∗*^ duplexes, based on a SVM model for plants. Similarly, another approach, miRdup [19], uses Random Forest algorithm integrated with Adaptive Boost (Adaboost) and can be used to detect the positions of miRNAs based on features from five lineages of cleavage sites on the miRNA:miRNA^*∗*^ duplexes. MiRPara [20] constructs two models to predict the location of mature miRNAs from genome-scale sequences for both plants and mammals. MirExplorer [26] is designed to predict pre-miRNAs and miRNAs with transition probability matrices and miRNA biogenesis vectors utilizing the Adaboost method for 16 species, while earlier methods, MIRcheck [27] and ProMir [5], are older tools for predicting mature miRNA candidates.

Although many methods are available for mature miRNA location prediction, they suffer from various limitations. For instance, many of these methods cannot be used to predict the miRtrons (miRNAs derived from the introns of protein-coding genes) [14], miRNAs without Drosha-mediated cleavage [21, 22, 24], or miRNAs that do not show strong evolutionary conservation [22]. In addition, since some approaches use a large number of features, it is difficult to achieve a possible biological explanation on which features contribute to the determination of the mature miRNA location [24]. Moreover, most of these prediction algorithms are based on early data resources [20, 26, 27], and several methods suffer from the inaccurate assumption that there is just one mature miRNA within a specific pre-miRNA transcript [5, 21, 24, 28]. Instead, only a few methods considered the double-stranded nature during miRNA maturation by integrating structure and sequence information. Thus, accurate prediction of mature miRNA locations remains a challenge [29].

In this study, we introduce a computational method, MatPred, which uses an RBF-SVM (radial basis function kernel/support vector machine) algorithm to predict the starting position of the mature miRNA in a pre-miRNA transcript. The parameters in the prediction model were trained using a set of experimentally validated mature miRNAs in the miRBase and further evaluated using a dataset that does not overlap with the training dataset. By systematically reviewing genomic features that can potentially affect the location of mature miRNAs, a more biologically relevant feature set was selected that includes the length of the mature and precursor miRNA transcript, the distances from the stem loop, and the minimum free energy of the RNA complex. More importantly, we innovatively design a series of features that represent the nucleotide and structure identities of each nucleotide for both miRNA:miRNA^*∗*^ complex and flanking regions. Comparison with existing tools suggests that MatPred achieved the highest prediction accuracy, according to the annotation documented in the latest version of miRBase (version 20).

## 2. Materials and Methods

### 2.1. Training and Test Datasets

Our training and test datasets were derived from 871 human pre-miRNAs documented in miRBase. These pre-miRNAs contained standard hairpin structures with single stem loops. In order to capture the features associated with experimentally validated starting positions of the mature miRNA molecules in the pre-miRNA sequences, we randomly selected 671 pre-miRNAs as our training dataset and further created two test sets, named Test set 1 and Test set 2, each containing 100 pre-miRNAs. In particular, Test set 2 is randomly selected from the newest dataset that only belongs to version 20. The list of training and test miRNA datasets can be found in Supplementary Table 1 in Supplementary Material available online at http://dx.doi.org/10.1155/2015/546763. In the training set (671 pre-miRNAs), the starting position of the experimentally validated mature miRNA was defined as the positive dataset, while all the other putative positions were considered the negative dataset. Overall, our training set contained 671 and 11,677 positive and negative data points, respectively.

### 2.2. RNA Secondary Structure Prediction

The secondary structure of the pre-miRNA was predicted using RNAfold [31], with default parameters. Since our tool, MatPred, was designed to identify mature miRNAs from experimentally identified novel pre-miRNAs, we did not use the secondary structure feature included in miRBase, thus allowing generalization of our algorithm.

### 2.3. Mature miRNA Loci within the Pre-miRNA Structure

**Figure 1:**
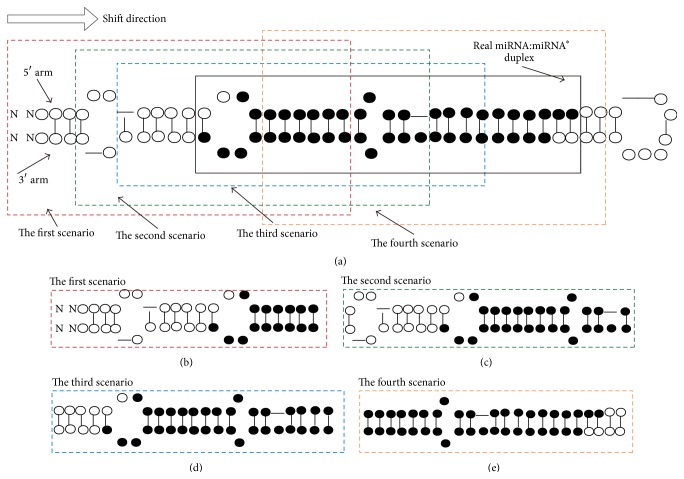
Definition of duplex window. (a) Four scenarios associated with duplex window selection. The solid black dot represents the nucleotides of the known mature miRNA. The hollow circles represent the nucleotides that are not part of the mature miRNA in the pre-miRNA transcript. All boxes represent duplex windows. The black box represents the known miRNA:miRNA^*∗*^ duplex. The four colored dashed boxes represent four scenarios while defining duplex window. (b)–(e) Detailed schematics corresponding to each scenario.

As shown in Figure 1, the goal of our algorithm was to identify the precise location of mature miRNAs (black circles) inside pre-miRNA structures. Without losing generalization, the mature miRNAs were assumed to be 22 nt in length. A sliding window scanning approach was taken by evaluating which nucleotide position was the most likely starting position of a box containing the mature miRNA:miRNA^*∗*^ duplex. Such a sliding box is defined as a duplex window.

Based on the known theory of miRNA biogenesis, the starting position of the duplex window is design as follows: the start position of mature miRNA on the 5′ arm shifted 2 nucleotides to the left [20]. The ending position of this window was the ending position of the mature miRNA on its 5′ arm, as extracted from the predicted secondary structure of the pre-miRNA. In addition, the starting and ending positions could not be “-” (i.e., a missing nucleotide, which would create a bulge structure with no paired base).

While searching for the duplex window (i.e., the mature miRNA location), hypothetically, four different scenarios could emerge (as shown in the dashed boxes in Figure 1(a)).

**Scenario 1 .**

The mature miRNA locates at the first nucleotide of the pre-miRNA. In this case, the duplex window will include 2 nucleotides before the starting position of 5′ arm in the pre-miRNA (Figure 1(b)).

**Scenario 2 .**

When the ending position base of 5′ arm in the duplex window was “-”; it will be right-shift by 1 nucleotide excluding “-” (Figure 1(c)).

**Scenario 3 .**

When the starting position character of the 5′ arm in the duplex window is “-”, the duplex window would be right-shifted by 1 nucleotide. The ending position would be dependent on the last nucleotide of the mature miRNA on its 5′ arm, which would be 22 nt from the preshifted position (Figure 1(d)).

**Scenario 4 (standard scenario).**

When both starting and ending positions are nucleotides, the defined window will be specified as duplex window (Figure 1(e)).

### 2.4. Features Describing the Duplex Window

We designed 94 features to describe each duplex window, including position-specific structure features, distances to the stem loop, and minimum free energy characterizing the stability of the RNA molecule.

*Duplex Window Region*. Duplex window region is a window which contains the mature miRNA:miRNA^*∗*^ duplex. For each nucleotide in the duplex, we assigned one of 9 values to describe its sequence-structure characteristics, Ap, Cp, Gp, Up, A-, C-, G-, U-, and - -, where A, C, G, and U indicate nucleotide identity and p and “-” denote pairing or not. If the 5′- and 3′-arms of the nucleotide sequences in the duplex window perfectly match, this region would contain 48 nucleotides. However, in order to accommodate bulge structures (“-”), we devoted 60 features to characterize the position-specific structure. If the total nucleotide number in the duplex window was less than 60, the unused allocation was assigned to be 0 (data missing). 

**Figure 2:**
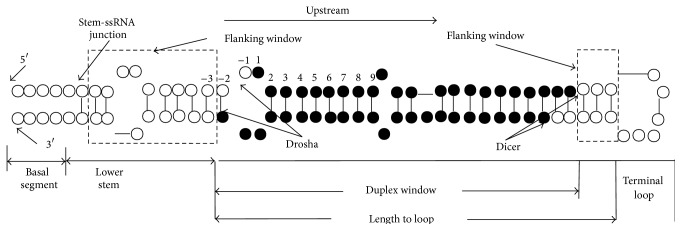
Schematic of pre-miRNA compartments. According to Han et al. [32], we divided the pre-miRNA hairpin loop into four main regions: basal segment, lower stem, miRNA:miRNA^*∗*^ duplex, and the terminal loop. The black circles represent the miRNA:miRNA^*∗*^ duplex which we extracted using a flexible sliding window of 22 nt nucleotides. The dashed boxes represent flanking regions. The hollow circles represent the nucleotides of the immature miRNA in the pre-miRNA.

*Lower Stem Loop (Figure 2)*. During miRNA biogenesis, the stem-ssRNA junction in the lower stem loop is often considered to determine the position of Drosha-processing sites, a key feature for localizing the mature miRNA in the pre-miRNA complex. Such junctions often locate ~11–13 nucleotides upstream of the duplex window [32]. In addition, the ~6–9 nucleotides flanking the mature miRNA usually include an internal loop and tend to have a specific sequence motif [33]. To capture such features, we extracted 18 sequence-structure characteristics of the upstream lower stem loop. Similarly, 6 additional features were assigned to the 3 pairs of nucleotides downstream the duplex window. 

*Minimum Free Energy*. Minimum free energy is important for RNA stability. Under a certain temperature, RNA molecules reach thermodynamic equilibrium for having the minimum free energy and forming the most stable structure through adjusting the conformation. The duplex window usually has stable double-stranded structures [32, 34], and it is considered to have the minimum free energy. Usually, the most stable structure is observed on the duplex window plus 3 nt upstream of the Drosha-processing sites, while 6 nt upstream of that (9 nt upstream of the Drosha-processing site) is the most unstable region. In addition, for the mature miRNA in the 3′ arm, the 19-20th nucleotides have the highest free energy and thus the most instability. Meanwhile, within the miRNA of the 5′ arm, the 12th base pair is relatively unstable [32]. In order to capture these known and other unknown characteristics regarding minimum free energy, we designed 5 measurements for different regions relative to the duplex window, including MFE1 (duplex window), MFE2 (duplex window + 3 bp upstream of Drosha site), MFE3 (duplex window + 5 bp upstream of Drosha site), MFE4 (duplex window + 9 bp upstream of Drosha site), and MFE5 (duplex window + 3 bp downstream of end of duplex window). In addition, 4 other features were designed to measure the differences in minimum free energy for various regions, MFE6 = MFE2 − MFE1, MFE7 = MFE3 − MFE2, MFE8 = MFE4 − MFE3, and MFE9 = MFE5 − MFE1. In addition, we also used the distance from the beginning of the duplex window to the terminal loop as one feature. Overall, 94 features were used to evaluate one putative duplex window.

### 2.5. RBF-SVM

Due to its superior performance on prediction accuracy, we chose RBF-SVM (support vector machine with the radial basis function kernel as the decision function) to identify the mature miRNA location. The parameters in the model are determined using a standard support vector classification (SVC) algorithm.

Given the training data, positive data *χ*_*i*_ ∈ *S*, *i* = 1,…, *m*, and negative data *χ*_*j*_ ∈ *S*, *j* = 1,…, *n*, and for each vector of training data corresponding class label *Z*_*i*_ ∈ {1, −1}, *α*_*i*_ is the coefficient that must be determined. SVC solves the following optimization problem:

(1) $$\begin{array}{c} g\left(\;\chi \;\right)=\overset{N}{\underset{i=l}{\sum }}Z_{i}\alpha_{i}k\left(\;\chi ,\chi_{i}\;\right)+\omega_{0}, \end{array}$$

where *χ*_*i*_ ∈ *S*, *i* = 1,…, *m*, and *χ*_*j*_ ∈ *S*, *j* = 1,…, *n*, represent data points in the *m* positive and *n* negative training dataset, respectively, and *α*_*i*_ is coefficient to be learnt (0 ≤ *α*_*i*_ ≤ *c*).

*k*(*χ*, *χ*_*i*_) is the RBF kernel; this data classification method has been applied to several biological problems [35] and is an effective method to map data onto an infinite-dimensional feature space. The RBF kernel function is as follows:

(2) $$\begin{array}{c} k\left(\;\chi_{i},\chi_{j}\;\right)=e^{-\gamma {\left\|\chi_{i}-\chi_{j}\right\|}^{2}}. \end{array}$$

The penalty parameter *c* and the RBF kernel parameter *γ* are implemented using the grid tool in the libSVM library [36].

This classifier offers the mature miRNAs candidates for the 5′ and 3′ arms. Every candidate is shown with two probabilities of belonging to any one category (positive or negative).

### 2.6. Prediction Outcome and Evaluation

For every nucleotide position in the pre-miRNA, a likelihood score will be calculated using the SVM model, based on the 94 features that are associated with the duplex window starting from its position. The accuracy of the model prediction is evaluated by comparing the distance between the predicted miRNA starting nucleotide from experimentally validated starting nucleotide on the 5′ arm; such distance is defined as position deviation (*D*(*x*)), where 0 means perfect prediction. A smaller position deviation indicates better prediction outcome.

## 3. Results and Discussions

### 3.1. Result

#### 3.1.1. Model Performance

We tested the performance of MatPred using two separate datasets (each contains 100 pre-miRNAs) that are independent of training set. Specifically, Test set 2 is exclusively selected from pre-miRNAs that are in miRBase V20, but not in V19. This test is designed to examine whether MatPred is capable of detecting newly discovered miRNAs.

**Figure 3:**
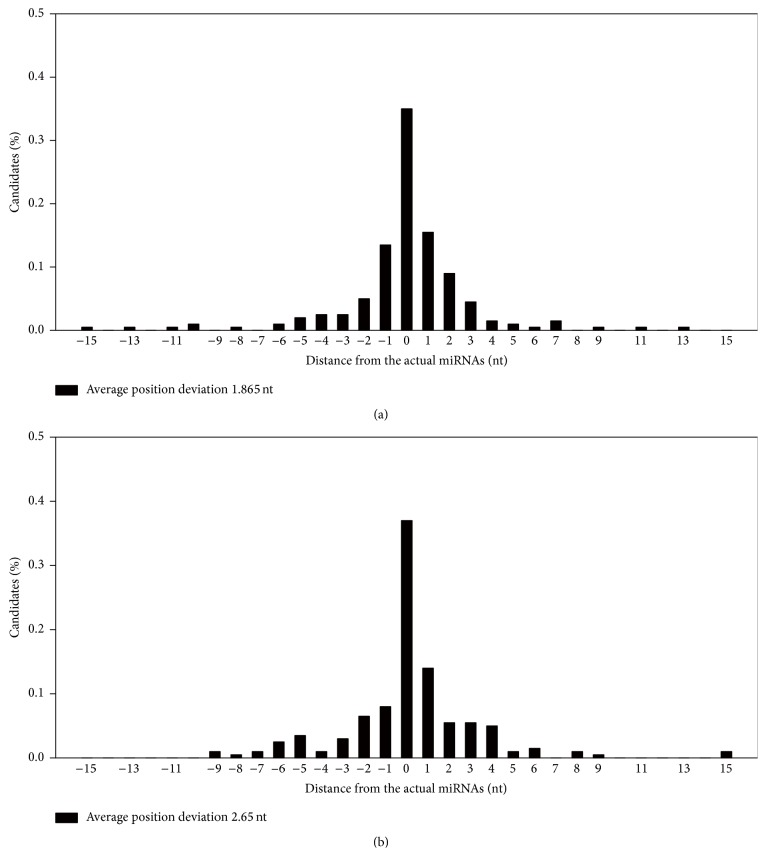
Distribution of the distances between predicted and known starting sites of mature miRNAs. (a) Test set 1 (randomly selected from nontraining dataset) and (b) Test set 2 dataset (selected from newly discovered miRNAs that are documented exclusively in miRBase V20).

As shown in Figure 3 and Table 1, MatPred achieved fairly good prediction accuracy. In Test set 1, 35% of predicted mature miRNA loci matched perfectly with the experimentally validated starting nucleotides. In addition, predicted starting positions of 92% and 97% of miRNAs are within 5 nt and 10 nt of the experimentally validated positions, respectively. The analysis on Test set 2 dataset achieved similar prediction accuracy (Figure 3 and Table 1). This suggests that MatPred can be used to predict locations of newly discovered miRNAs. The overall position deviations, distance distribution (DS), for the two test datasets are 1.9 and 2.7 nucleotides, respectively. Figure 3 shows the average distance (AD) distributions over the Test 1 and test new datasets, illustrating the performance of MatPred for predicting the mature miRNA location.

#### 3.1.2. Comparisons with Other Methods

In order to compare the performance of our method with other published algorithms, we selected three methods that integrated a variety of sequence and structure features, MaturePred [25], MatureBayes [23], and miRdup [19]. These three algorithms are publicly available and actively maintained. In order to ensure the fair comparison with other methods, we used Test set 2, which contains only newly discovered miRNAs that are not documented in miRBase V20. This will guarantee that the entries in our test set do not overlap with the records in the training set of other algorithms and therefore avoid bias.

**Figure 4:**
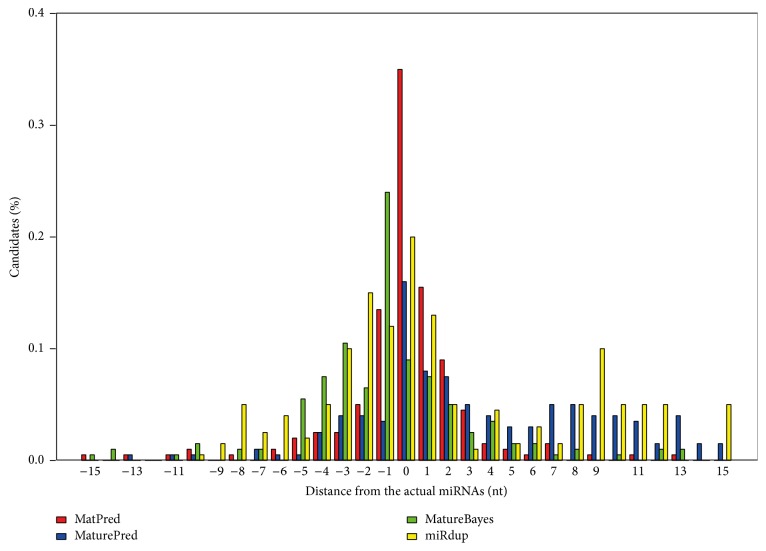
Distribution of distances between known mature miRNA starting sites and the ones predicted by MatPred, MaturePred, MatureBayes, and miRdup, based on newly discovered miRNAs (Test set 2).

As shown in Figure 4 and Table 2, MatPred significantly outperformed the other three methods. The average position deviation, DS, for MatPred is 2.65 nt, as in comparison to 4.0 nt, 5.0 nt, and 3.0 nt for MaturePred, MatureBayes, and miRdup, respectively. In addition, our algorithm predicted 37% of the starting positions that coincided with those of the known miRNAs, significantly better than the 16%, 7%, and 19% predicted by MaturePred, MatureBayes, and miRdup, respectively.

Comparison between Test set 1 and other algorithms showed similar trend: MatPred outperformed all the other tools for prediction accuracy. Briefly, MatPred predicted that 35% of the starting positions coincided with those of known miRNAs, which is significantly better than the 16%, 9%, and 20% as predicted by MaturePred, MatureBayes, and miRdup, respectively. In addition, the average position deviation between predicted and known miRNA starting site is 1.865 nucleotides, while the respective values for MaturePred, MatureBayes, and miRdup were 5.603 nt, 4.22 nt, and 4.045 nt.

In order to compare the performance of our method for precise identification of the position of mature miRNAs with other published algorithms, we selected hsa-miR-6855, hsa-miR-6764, hsa-miR-7114, and hsa-miR-6894 which are the newly discovered pre-miRNA in miRBase V20 and hsa-miR-504 which is known pre-miRNA and experimentally verified having one miRNA in miRBase V19 as the test data. For the novel pre-miRNAs, the identification start positions of hsa-miR-6855-5p, hsa-miR-6764-5p, hsa-miR-7114-5p, and hsa-miR6894-5p using our method were supported by present reports [37, 38]. MatureBayes, MaturePred, and miRdup identify the start position of hsa-miR-6855-5p with −1 nt, −1 nt, and 1 nt deviation, hsa-miR-6764-5p with 2 nt, 1 nt, and 1 nt deviation, hsa-miR-7114-5p with 3 nt, 2 nt, and 2 nt deviation, and hsa-miR6894-5p with 2 nt, −1 nt, and −1 nt deviation. For the known pre-miRNAs, our method discovers the new miRNA and identifies the start position of hsa-miR-504-3p which is proved by the present reports [37, 39]. And MatureBayes, MaturePred, and miRdup predicted its position with −3 nt, −1 nt, and 3 nt deviation.

### 3.2. Discussions

Although the secondary structure properties of pre-miRNA transcripts have been long exploited in miRNA biology, they have only been effectively used in some mature miRNA prediction methods [19, 23, 30]. In this study, we report a new prediction algorithm, MatPred, for predicting the location of mature miRNAs within the novel pre-miRNA transcripts, based on RBF-SVM. The major purpose of our algorithm is to identify mature miRNA loci from precursor miRNA sequences, not to distinguish pre-miRNAs from pseudo-pre-miRNAs which include sequence hairpin structures derived from the 3′UTR regions. Therefore, the noncanonical mature miRNA loci will serve as a base for false prediction.

Based on the secondary structure prediction for the pre-miRNA transcripts, MatPred generates a feature list that integrates the RNA secondary structure properties of each nucleotide within and flanking the mature miRNA region. In addition, several other features that are highly relevant to miRNA biogenesis were selected, including length of mature and pre-miRNA, distances from stem loop, and minimum free energy. Such features not only described the base-pairing structures of miRNA:miRNA^*∗*^ complex but also captured RNA stability characteristics that associate with different regions on the pre-miRNA transcripts; for instance, a bulge at lower stem region (5′-flanking region of the duplex window in Figure 2) may help to create higher free energy for Drosha cleavage.

As evaluated by two independent datasets, our model showed significant improvement on the prediction accuracy compared to several existing methods. Remarkably, ~35% predictions accurately reported the known locations of mature miRNAs, and over 90% of the predicted positions were within 5 bp of known sites.

It should be noted that our prediction is based on static pre-miRNA sequences presented in the miRBase database, which is derived from the reference sequence assembly. This model does not consider the genetic variations in the human population, which may affect the accuracy of the prediction. In addition, it has been widely reported that mature miRNAs have significant end-heterogeneity. This may further underestimate the accuracy evaluation on our method.

## 4. Conclusion

In conclusion, our method suggests that the biologically relevant features extracted from extending duplex windows, combined with RBF-SVM, generate an effective classifier for mature miRNA location identification. Such modeling could provide key insight into miRNA processing and biogenesis.

## Supplementary Material

Supplementary Table 1: proposes the training and test datasets.

## Figures and Tables

**Table 1:** Cumulative percentage of position deviation, DS, between predicted and known mature miRNA starting site.

DS	±0	±1	±2	±3	±4	±5	±6	±7	±8	±9	±10	AD
Test 1	0.35	0.64	0.78	0.85	0.89	0.92	0.94	0.95	0.96	0.96	0.97	1.865
Test 2	0.37	0.59	0.71	0.79	0.85	0.90	0.94	0.95	0.96	0.98	0.98	2.650

**Table 2:** Cumulative percentage of position deviation between known mature miRNA starting site and predicted ones based on four prediction algorithms.

DS	±0	±1	±2	±3	±4	±5	±6	±7	±8	±9	±10	AD
MatPred	0.37	0.59	0.71	0.80	0.86	0.90	0.94	0.95	0.97	0.98	0.98	2.650
MaturePred	0.16	0.29	0.36	0.42	0.49	0.58	0.64	0.74	0.82	0.85	0.88	4.005
MatureBayes	0.07	0.28	0.47	0.64	0.71	0.77	0.81	0.82	0.85	0.86	0.86	5.101
miRdup	0.19	0.42	0.59	0.65	0.74	0.82	0.86	0.91	0.94	0.96	0.98	3.004
