# Supplementary figures and images for: MatPred: Computational Identification of Mature MicroRNAs within Novel Pre-MicroRNAs
Source: Biomed Res Int. 2015 Nov 23;2015:546763. doi: 10.1155/2015/546763 (PMC4670854; doi:10.1155/2015/546763)

A

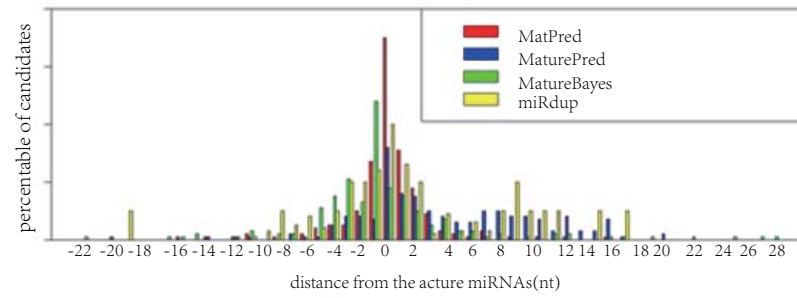

B

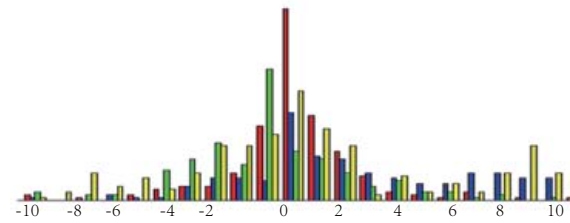

Supplement: Supplementary file 1 — Supplementary Table 1: proposes the training and test datasets. [file 546763.f1.pdf]
